# The effect of atomoxetine on random and directed exploration in humans

**DOI:** 10.1371/journal.pone.0176034

**Published:** 2017-04-26

**Authors:** Christopher M. Warren, Robert C. Wilson, Nic J. van der Wee, Eric J. Giltay, Martijn S. van Noorden, Jonathan D. Cohen, Sander Nieuwenhuis

**Affiliations:** 1 Institute of Psychology, Leiden University, Leiden, Netherlands; 2 Leiden Institute for Brain and Cognition, Leiden University, Leiden, Netherlands; 3 Department of Psychology and Cognitive Science Program, University of Arizona, Tucson, Arizona, United States of America; 4 Department of Psychiatry, Leiden University Medical Center, Leiden, Netherlands; 5 Department of Psychology, Princeton University, Princeton, New Jersey, United States of America; 6 Princeton Neuroscience Institute, Princeton University, Princeton, New Jersey, United States of America; VU University Medical Center, NETHERLANDS

## Abstract

The adaptive regulation of the trade-off between pursuing a known reward (exploitation) and sampling lesser-known options in search of something better (exploration) is critical for optimal performance. Theory and recent empirical work suggest that humans use at least two strategies for solving this dilemma: a directed strategy in which choices are explicitly biased toward information seeking, and a random strategy in which decision noise leads to exploration by chance. Here we examined the hypothesis that random exploration is governed by the neuromodulatory locus coeruleus-norepinephrine system. We administered atomoxetine, a norepinephrine transporter blocker that increases extracellular levels of norepinephrine throughout the cortex, to 22 healthy human participants in a double-blind crossover design. We examined the effect of treatment on performance in a gambling task designed to produce distinct measures of directed exploration and random exploration. In line with our hypothesis we found an effect of atomoxetine on random, but not directed exploration. However, contrary to expectation, atomoxetine reduced rather than increased random exploration. We offer three potential explanations of our findings, involving the non-linear relationship between tonic NE and cognitive performance, the interaction of atomoxetine with other neuromodulators, and the possibility that atomoxetine affected phasic norepinephrine activity more so than tonic norepinephrine activity.

## Introduction

The explore-exploit dilemma refers to the question, when deciding what to do, of whether it is better to stick with a known quantity, or explore unknown options that may yield less or more value [1–4]. When people make choices in an environment that includes multiple options of uncertain value, optimal performance requires a balance of both behaviors: exploitation of high-value options when they are known, and exploration of lesser known options to potentially discover better choices. Solving the dilemma requires determining when and how to explore versus exploit [5]. Two strategies that have been most prominently discussed in theoretical accounts of the explore-exploit dilemma are directed exploration [6–8] and random exploration [9–12]. Directed exploration involves making choices specifically to gain information about the value of an unknown option, and balancing the value of that information (the “information bonus”) against the expected reward value that a known option would yield. A carefully calibrated information bonus involves taking into account the mean and variance of the distribution of expected values for each option, as well as the number of choices that can be made within a set reward structure. Mathematical analyses indicate that an optimal decision maker would use directed exploration [5], but directed exploration is computationally demanding and can become untenable in more complicated, uncertain and ecologically valid circumstances [4,6,7]. In contrast, random exploration offers an alternative that is computationally very easy—merely relying on a portion of random choices to discover valuable options—and is less vulnerable to being influenced by outlier observations that can produce a misleading information bonus[4][4].

Wilson and colleagues [4] suggested, based on earlier proposals [12,13], that random exploration may be governed by baseline norepinephrine (NE) levels. The noradrenergic system has widespread projections throughout the central nervous system, where the release of NE increases the responsivity of target neurons, thus exerting a global influence on neural activity [14,15]. Transient increases in NE can be highly advantageous for task-relevant behaviour when applied at the right time, but high NE can also propagate the influence of noise and induce more variable behaviour when applied indiscriminately [12]. Accordingly, NE levels may govern the balance between exploitative, value-based choice and random exploration. Specifically, the adaptive gain theory [12] proposes that tonic increases in cortical NE levels from low to intermediate facilitate exploitative behavior, whereas tonic increases in NE levels from intermediate to high levels promote disengagement from current behaviors in the service of exploration, by increasing decision noise.

Animal studies have yielded some direct evidence for a role of the noradrenergic system in regulating the tradeoff between exploration and exploitation [16,17]. For example, Tervo and colleagues [17]examined the effect of optogenetic and pharmacogenetic manipulations of NE on behavioral choices of rodents faced with virtual competitors. Playing against computer algorithms developed to predict responses on the basis of past history, the rodents learned to exploit specific response patterns to maximize winnings against weaker opponents, but to abandon systematic response contingencies in favor of random responding against stronger opponents. Adoption of this random response mode could be facilitated by enhancing NE release and be counteracted by suppressing NE release in the anterior cingulate cortex. More recently, Kane et al. [18] have used designer-receptors (DREADDs) expressed in the LC [19,20] to demonstrate a causal relationship between increased tonic LC activity and disengagement from ongoing behavior in a foraging task.

Studies examining the role of NE in explore/exploit behavior in human participants have mainly relied on pupillometry as a non-invasive method of indexing endogenous fluctuations in NE levels [21]. One study found that baseline pupil size, a correlate of tonic NE levels [12,22–24], predicted task disengagement and the choice to abandon a current task in favor of one with a different reward structure [21]. In another study, Jepma and Nieuwenhuis [25] measured baseline pupil size in participants performing a four-choice gambling task with a gradually changing payoff structure, in which the trade-off between exploitation and exploration is a central component. Jepma and Nieuwenhuis showed that exploratory choices were preceded by a larger baseline pupil diameter (indexing higher NE levels), and that individual differences in baseline pupil diameter were predictive of a subject’s tendency to explore. The authors also fit a reinforcement learning model to the choice data and estimated for each subject the information bonus and the gain parameter (or inverse temperature) of the softmax decision rule, which indicates how closely decisions are constrained by the difference in estimated reward value among the four options. This gain parameter corresponds closely to the noise parameter of the logistic psychometric function used by Wilson et al. [4] and in the work reported below. Interestingly, individual differences in baseline pupil diameter correlated with the gain parameter but not with the information bonus, consistent with the idea that NE levels regulate random but not directed exploration. However, it is worth noting that most of the participants in this study were characterized by a negative information bonus, meaning that they tended to avoid instead of explore uncertain options. It is possible that these participants were ambiguity-averse and therefore avoided informative options. But perhaps more importantly, the informational value and expected reward value of the four options were confounded. Given that the pay-off structure of the four options changed gradually and slowly, the most informative options tended to be options with a relatively low reward. Similar correlations between pupil dilation and decision noise have also been found for decisions in other contexts, including perceptual decisions about the direction of moving dots [26] and gambling decisions in which the probabilities of rewards were known (i.e. not an explore/exploit gambling task) [27].

Although these indirect methods of examining the effect of NE on explore/exploit behavior provide some support for the proposed relationship, psychopharmacological manipulation provides a method for directly manipulating cortical NE levels to establish a causal role for NE in human explore/exploit behavior. One previous study [28] examined the effect of reboxetine, a selective NE transporter blocker, on explore/exploit behavior in two tasks previously used to demonstrate that baseline pupil diameter predicts task disengagement [21]and exploratory choices [25]. Jepma and colleagues [28]failed to find an effect of reboxetine in either task, despite finding significant effects of treatment on central and autonomous nervous system parameters. Below we will discuss some potential explanations for these null findings (see Discussion).

In the present study we administered atomoxetine and placebo to 22 healthy human participants in a double-blind crossover design and examined the effect of atomoxetine on independent measures of random and directed exploration, obtained using the Horizon Task developed by Wilson and colleagues [4]. Atomoxetine is a selective NE transporter blocker that is used to treat attention-deficit hyperactivity disorder [29–31]. It works by blocking the NE transporter, thus increasing extracellular levels of NE throughout the cortex [29]. Atomoxetine plasma concentrations remain elevated up to 9 hours after drug ingestion [30], and atomoxetine administration induces physiological effects including increased pulse rate, and increased salivary cortisol concentration for at least 3.5 hours after a single dose [32]. We expected to replicate the finding of Wilson and colleagues that humans use both directed and random exploration. Importantly, we hypothesized that atomoxetine administration would affect random, but not directed exploration.

## Results

### Horizon task

**Fig 1:**
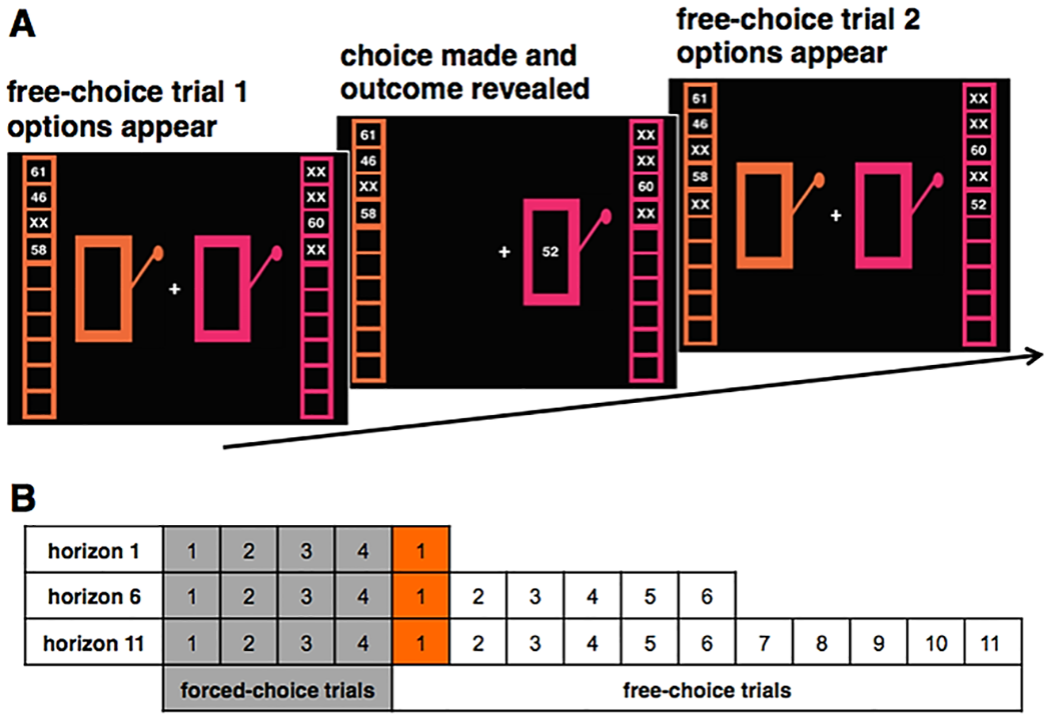
Task design. Panel A presents three screenshots showing events surrounding one free choice in a horizon 6 game. The first four choices were constrained to one of the two options in order to control the information available to the subject (A, left). The constraint was indicated by a white box surrounding the choice that the subject was forced to select in order to continue. The columns on each side of the screen showed the history of previous choices, and the number of choices remaining (empty boxes). The example shown represents the unequal information condition, because more is known about the left option than about the one on the right. When a choice was made (A, middle) the outcome value was revealed, and when the next option was presented (A, right) this outcome appeared in the history of the chosen option. Panel B gives a schematic of the different trial types in the three horizon conditions. The first free choice (colored orange) yielded the critical data analyzed here and in previous work [4].

The Horizon Task that participants performed in our study was developed by Wilson and colleagues [4] to examine the extent to which humans use directed versus random exploration to solve the explore-exploit dilemma. In this task participants played a series of games allowing them to choose between two options with an underlying reward structure that was fixed for each game, but unknown to the participants (Fig 1). A key feature of the task is that the number of choices the participants could make in a given game varied between games. This number, referred to as the horizon, is particularly important for studying exploration, because if participants only get one choice within a set reward structure (i.e., a horizon of one), there is no way for them to benefit from what might be learned. In contrast, if a subject has six choices (i.e., a horizon of six), then there is value in spending some initial choices learning about lesser-known options because the later choices could benefit from this information. By manipulating the horizon, we were able to look at the degree to which directed and random exploration changed from a horizon of one favoring exploitation (baseline) to a longer horizon of six that should motivate exploration. By taking into account baseline choice behaviour in horizon 1, this method controls for individual differences in approach or avoidance tendencies toward ambiguity [33,34] and behavioural variability [4].

A second key feature of the Horizon Task is that the information known about each option and the reward value of that option are decorrelated. A confound in previous work has been that participants tend to gain more information about high-value options because they select high-value options more often [4]. In the Horizon Task, we controlled the first four selections that participants made in each horizon condition, and therefore the available information about the low- and high-value options before participants made their first free choice. We factorially manipulated reward value and information, such that participants could have equal information about the low- and high-value options (two choices from each), or unequal information (three choices from one option, one choice from the other; see Materials and methods). This way, the Horizon Task enabled us to extract independent measures of directed and random exploration.

**Fig 2:**
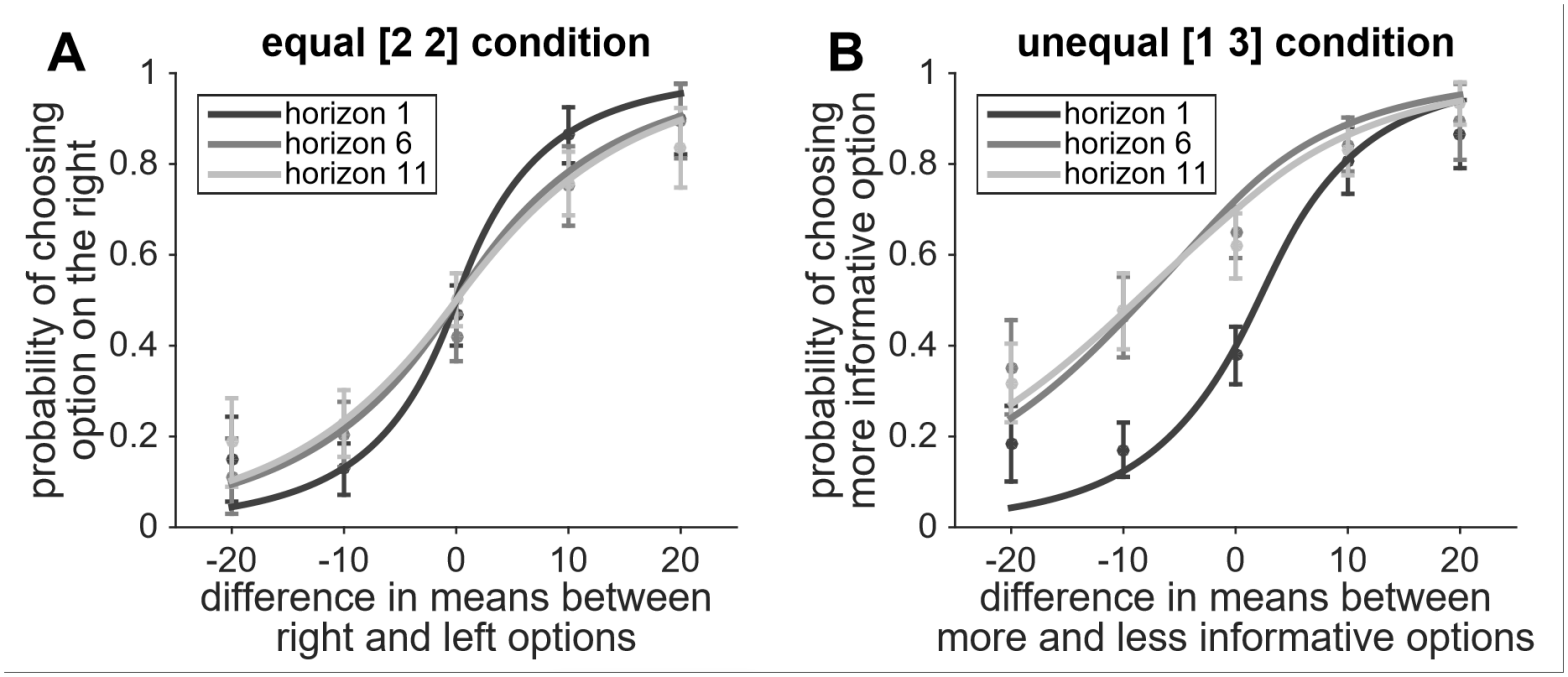
Choice curves as a function of horizon and information condition. When exploration is motivated by a long horizon, the slope of the curve gets less steep (more decision noise), and the entire curve shifts toward the more informative option (in the unequal information condition), illustrating how the more informative option has value that offsets taking a lower reward in order to explore. Note error bars are 95% confidence intervals.

Following Wilson and colleagues [4], we quantified directed and random exploration by fitting a psychometric function to participants’ choices, that described the frequency of choosing the more informative option as a function of the difference in expected reward value between the more and less informative options (see Fig 2a and 2b). For each participant, we determined the value of the bias and slope of a logistic function that best fit that participant’s pattern of choices for each experimental condition, and used these values as estimates of directed and random exploration, respectively. The bias term described the indifference point condition (see Fig 2a); that is, the difference in value between the more and less informative options (along the X axis) at which participants were equally likely to choose either option (Y = 0.5). A negative indifference point suggests that the higher expected reward value of the less informative option is offset by the informational value of the more informative option. This offset from zero was used as an estimate of the information bonus, and directed exploration was operationally defined as the change in information bonus from horizon 1 to longer horizons. The slope of the function at the indifference point (sometimes referred to as its gain) was used to estimate decision noise (see Fig 2a and 2b); that is, the strength of the relationship between the subject’s choice and the difference in expected value between the two options. When there is less decision noise, subjects are heavily influenced by the expected values of the two options, but as decision noise increases this relationship is weakened such that the choice curve gets less steep. Random exploration was operationalized as the difference in slopes from horizon 1 to longer horizons. We expected to replicate the finding of Wilson and colleagues that humans use both directed and random exploration by demonstrating increases in information bonus and decision noise, respectively, in games with longer horizons (relative to the horizon 1 baseline). Importantly, we hypothesized that atomoxetine administration would affect random, but not directed exploration, by increasing decision noise in the longer horizon conditions.

### Model-free analysis of exploration

Fig 2 shows the probability of making a specific choice as a function of the difference in value between the two options for the equal information condition (Fig 2a) and the unequal information condition (Fig 2b), averaged across all participants and both treatment conditions. To get a first indication of whether the manipulations of horizon (1, 6, or 11) and available information (equal or unequal) affected exploratory behavior, we examined the proportion of games in which participants, on the first free-choice trial, chose the option with the lower expected value (an exploratory choice). A repeated-measures ANOVA yielded a main effect of horizon, indicating that participants were more likely to pick the choice with the lowest value when they were in the horizon 6 and horizon 11 conditions (M_6_ = .39, M_11_ = .38) than in the horizon 1 condition (M_1_ = .33, *F*(2, 40) = 7.22, *P* < 0.01); and a marginally significant effect of information condition, indicating that participants were more likely to pick the lower-value option in the unequal information condition (M_unequal_ = .38; M_equal_ = .35; *F*(1, 20) = 4.12, *P* = 0.056. These model-free findings are suggestive of horizon- and information-dependent exploration.

### Model-based analysis of directed exploration

**Fig 3:**
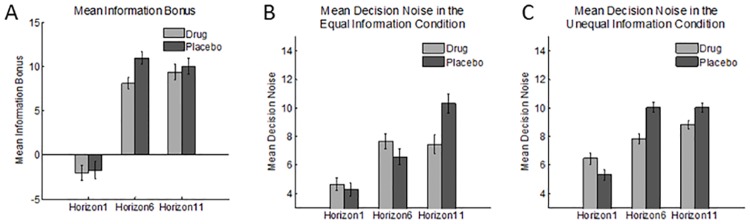
Subject-level Bayesian estimates of the information bonus (a) and decision noise (b, c) for each treatment. Both the information bonus and decision noise were markedly increased at longer horizons compared to baseline (horizon 1). Treatment reduces the increase in decision noise from baseline to later horizons in both the equal information condition (b), and the unequal information condition (c) Error bars reflect 95% confidence intervals.

Our model-based analysis allowed us to estimate distinct measures of directed (information bonus) and random exploration (decision noise). The subject-level Bayesian estimates of the means for each task condition and treatment are displayed as bar graphs in Fig 3, for the information bonus (Fig 3a) and for decision noise (Fig 3b and 3c). The median group-level estimates of means and standard deviations are provided in Table 1.

**Table 1:** Group-level parameter estimates of decision noise.

	**Equal Information**					
	**Horizon 1**		**Horizon 6**		**Horizon 11**	
	**Placebo**	**Drug**	**Placebo**	**Drug**	**Placebo**	**Drug**
Mean	4.162	4.388	6.369	7.180	9.562	7.121
SD	2.129	0.591	1.652	0.492	0.502	0.934
	**Unequal Information**					
	**Horizon 1**		**Horizon 6**		**Horizon 11**	
	**Placebo**	**Drug**	**Placebo**	**Drug**	**Placebo**	**Drug**
Mean	5.217	6.214	9.79	7.601	9.665	8.702
SD	2.037	0.843	2.75	1.336	1.510	7.042

Wilson and colleagues demonstrated directed exploration by showing that the information bonus on the first free-choice trial was significantly larger for horizons 6 and 11 than for horizon 1 (Wilson et al., 2014, Supplementary Material). We replicated this effect, *F*(2,126) = 6.57, *P =* .002 (Figs 2a and 3a; Table 1*)*. This means that participants assigned greater value to the more informative option when they knew they would have additional choices that could benefit from what was learned.

We anticipated that atomoxetine would affect only random exploration, and thus expected no effects of treatment on the size of the information bonus. Consistent with this, there was no effect of treatment, *F*(1,126) = .28, *P* = .60 and no interaction of treatment with horizon, *F*(2,126) = .25, *P* = .78 (Fig 3a*)*. Thus, atomoxetine did not affect directed exploration.

### Model-based analysis of random exploration

Wilson and colleagues also found evidence for random exploration, indexed by an increase in decision noise with horizon: curves for horizons 6 and 11 were less steep than for horizon 1 (Wilson et al., 2014, Supplementary Material). We replicated this effect, *F*(2,252) = 53.33, *P* < .001. We also replicated the effect of information condition on decision noise, *F*(1,252) = 20.70, *P* < 0.001 (Figs 2a, 2b, 3b and 3c; Table 1), indicating that decision noise was larger in the unequal information condition. The interaction between horizon and information condition was not significant, *F*(2,252) = 1.02, *P* = .36. These results indicate that when exploratory behavior was motivated by a longer horizon, or by having less information about one option, decision noise increased on the first free-choice trial and thus decisions became less related to the difference in expected reward value of the two options, promoting exploration.

In addition, there was evidence of treatment effects on decision noise. Although the main effect of atomoxetine on decision noise was only marginally significant, *F*(1,252) = 3.71, *P* = 0.055, there was a significant interaction of treatment with horizon, *F*(2, 252) = 4.73, *P* < .01, indicating that the increase in decision noise from baseline to longer horizons was smaller in the drug condition than in the placebo condition (Fig 3b and 3c; Table 1). This finding represents an effect of treatment on random exploration, whereby atomoxetine reduced random exploration. This interaction was partly driven by a trend towards a treatment-induced increase in decision noise for horizon 1, the baseline condition, *F*(1, 84) = 3.38, *P* = 0.07), which may reflect an effect of atomoxetine on ambiguity aversion. Finally, there was a significant three-way interaction of treatment with horizon and information condition, *F*(2,252) = 5.08, *P* < .01, which indicated that the effect of treatment on random exploration was more consistent in the unequal information condition than in the equal information condition (Fig 3b and 3c; Table 1), perhaps because there was more room for a drug-related decrease in decision noise in the unequal information condition.

### Treatment effects on reaction time

In previous work, high tonic NE activity has been associated with relatively slow and more variable reaction times [21,35]. We analyzed mean reaction time and reaction time variability for the first trial after the four forced-choice trials. Reaction time was numerically faster in the atomoxetine condition (1126 ms vs. 1136 ms) although this difference was not significant (*P* = .91). Similarly, there was no effect of treatment on reaction time variability (*P* = .69), although numerically variability was lower in the atomoxetine condition. Similar results were obtained when we analyzed data from all free-choice trials. Although these findings should be interpreted with care, they suggest that atomoxetine did not increase reaction time, nor make it more variable, as would be expected had atomoxetine promoted a shift toward higher tonic NE activity.

### Treatment effects on task performance

We analyzed task performance in terms of total reward earned as well as the percentage of choices participants made that were “correct”, in that they selected the option associated with the highest mean reward. Although atomoxetine was associated with higher overall reward (52.4 vs. 52.2, *P* = .32) and more correct choices (67.9% vs 67.7%, *P* = .91), these differences were not significant.

## Discussion

We used atomoxetine as a pharmacological manipulation of NE levels and examined the effect on directed and random exploration. As predicted, we found that atomoxetine had a selective and significant impact on decision noise. However, contrary to our expectations it diminished rather than augmented the horizon-dependent increase in decision noise associated with random exploration. Atomoxetine did not affect directed exploration. In addition, we found no effect of treatment on reaction time, nor on task performance. Taken together, these results suggest that pharmacological manipulation of NE levels affects random, but not directed exploration, as proposed by Wilson and colleagues [4]. However, the treatment-induced attentuation of random exploration is at best difficult to interpret with respect to the theoretical predictions.

Perhaps the simplest explanation for this finding is that NE does not boost random exploration, as claimed by adaptive gain theory [12], but rather attenuates exploration by reducing decision noise. Though such an interpretation is inconsistent with the substantial literature upon which our predictions were based [3, 16, 17; 21,; 25;], it is consistent with findings in the bird song literature in which decreased NE levels (brought about by the use of NE specific neurotoxin DSP-4) were associated with greater variability in song production [36].

A second explanation is that three hours into the test session (see Materials and methods) participants started the task in a fatigued state, characterized by relatively low, below-optimal baseline NE levels and increased decision noise. Atomoxetine could have counteracted this by increasing NE toward the intermediate levels that are associated with optimal cognitive task performance [12], thus reducing decision noise. More research is needed to examine how the amount of decision noise varies between low, intermediate and high NE levels.

A third explanation is that atomoxetine may not have produced the intended effect in the cortex. Although it is commonly assumed that atomoxetine increases tonic (i.e., baseline) levels of extracellular NE, this assumption is based mainly on microdialysis studies of NE reuptake inhibitors in animal models [37]. However, microdialysis has limited temporal resolution, and cannot distinguish whether changes in dialysate NE levels, obtained over many minutes of sampling, are due to changes in tonic versus phasic NE release, or both. Therefore, it is uncertain whether our drug manipulation increased NE levels in the tonic, indiscriminate manner characteristic of distractible, exploring animals [12]. Indeed, the behavioral data provide preliminary evidence that atomoxetine administration did not promote higher tonic NE activity, in contrast to our assumption. Based on the work of Gilzenrat and colleagues [21,35], we would expect that if atomoxetine promoted a shift toward high tonic NE activity, then atomoxetine administration should be associated with slower and more variable reaction times. However, we observed non-significant effects in the opposite direction. This raises the question, if atomoxetine did not increase tonic NE activity, then what did it do?

One possibility is that the effect of atomoxetine on other neuromodulators played a role in producing these results. An important issue to consider is the non-specificity of atomoxetine action with regard to NE and dopamine [29]. In particular, it is well-established that atomoxetine increases prefrontal dopamine levels [29,38,39]). Frank and colleagues have reported a link between prefrontal dopamine and directed exploration [40–42]. In particular, they focused on the COMT gene, which plays a major role in dopamine degradation in the prefrontal cortex. They found that individual differences in directed exploration are associated with individual differences in the COMT gene[40], and further, that pharmacological inhibition of COMT leads to greater directed exploration [42]. In addition, patients with schizoprenia exhibit reduced directed exploration, which was predicted based on putative schizophrenia-related deficits in prefrontal dopamine function [41]. Based on this association between COMT and directed exploration, one might have predicted an atomoxetine-related increase in directed exploration. However, we did not observe any treatment-related changes in directed exploration in our study. This null effect should not be interpreted as evidence against a role of prefrontal dopamine in directed exploration. Indeed, it is possible that the effect of atomoxetine on prefrontal dopamine was small. Moreover, because the Horizon Task and the Clock Task of Frank and colleagues [40] have never been performed in the same subjects, it is not clear whether directed exploration as measured in the task of Frank and colleagues is the same as directed exploration probed by the Horizon Task.

A second possibility is that atomoxetine increased task-relevant phasic NE release, with or without a concomitant increase in tonic NE levels. Aston-Jones and Cohen [12] described firing profiles in the locus coeruleus ranging from relatively constant high-frequency firing (tonic mode, linked to exploratory behavior), to mostly low-frequency tonic firing with pronounced phasic bursts of high-frequency firing in response to motivationally salient events (phasic mode, associated with exploitative behavior). The outcome of the former activity would be sustained, high levels of NE in the cortex, an effect that atomoxetine administration was intended to produce. However, instead, atomoxetine may have amplified the temporal profile characteristic of the latter mode of activity. In line with this, Bari and Aston-Jones [43] found that atomoxetine administration increased the phasic-to-tonic ratio of LC responses. Physiological recordings from LC consistently demonstrate an inverse relationship between phasic firing and tonic LC activity [44,45, c.f. 46]). Thus, if atomoxetine augmented phasic firing, not only might it have left intact or even increased task-driven fluctuations in NE (thereby potentiating the processing of the most valuable or salient option), but it may have also directly or indirectly reduced tonic firing, thereby reducing decision noise and decreasing exploration, the result we obtained.

Our results seem inconsistent with Jepma et al.[28], who found no effect of the selective NE-transporter blocker reboxetine on random exploration in a four-choice gambling task. This apparent discrepancy may reflect several critical differences between the two studies. First, the gambling task of Jepma and colleagues was not designed to distinguish between directed and random exploration. As noted above, the informational value and expected reward value of the four options were confounded in a way that makes directed exploration difficult to detect. Second, the inclusion of horizon 1 in the present work allowed us to examine the *change* in exploration from horizon 1 to longer horizons, and thus control for individual differences in ambiguity aversion, a variable that was not taken into account by Jepma and colleagues. Third, Jepma et al. used a between-participants design, and thus had no baseline measure of exploratory behavior in the participants who received treatment. Although between-subject designs are less sensitive to practice effects, they have reduced power for detecting treatment effects. Finally, it is possible that atomoxetine and reboxetine, in the doses used, differ somewhat in their effects on NE release, LC firing activity, and the net effect of these actions [37].

Our work holds additional value in that we replicated the findings, reported by Wilson and colleagues [4], that humans tend to use both directed and random exploration in solving the explore-exploit dilemma. Moreover, our findings suggest that directed and random exploration have different neural substrates. This is consistent with recent findings suggesting that the two types of exploration have different developmental profiles [47] and that directed, but not random exploration, is dependent on the frontal pole [48]. Taken together, these findings are consistent with the idea that a hybrid of directed and random exploration is the most effective strategy for solving explore/exploit tasks when the limitations of human cognition are taken into account. Such a hybrid strategy attenuates the computational cost of purely directed exploration, while mitigating the probability of making a random response in favor of a clearly inferior option. A flexible hybrid, that adapts the level of random exploration employed to the level of uncertainty in the environment, could be implemented by changes in baseline NE levels [3,12,17,25,49]. The current experiment, with a modest sample size, provides preliminary evidence for this hypothesis. Future work, using larger sample sizes, or using optogenetics or direct measurements of baseline NE levels, may provide more definitive tests of this hypothesis.

## Materials and methods

### Participants

Twenty-two healthy volunteers (13 females) between 18 and 30 years (mean age: 22.0 ± 1.7 years) completed the experiment. All participants were undergraduate students at Leiden University. To capitalize on the duration of the drug effects, participants completed additional experiments during the same sessions, before carrying out the Horizon Task: a simple object classification task while lying in an MRI-scanner (reported in [50]) and a challenging 10-minute letter-identification task outside of the scanner (reported in [51]). Participants were paid 135 euros. All participants gave written informed consent. The study was approved by the ethics committee of the Leiden University Medical Centre.

No standardized screening instruments were used for pre-screening the participants. Physical health was assessed in the university hospital setting by a psychiatrist (MvN, EG, NvdW), consisting of a medical history and general physical examination that included a blood pressure measurement. We aimed to identify those who are at greater risk of somatic conditions or the development of side effects, in order to only select those participants who were at low risk for complications. Exclusion criteria included: current use of prescription medication, a history of psychiatric illness, cardiovascular disease, renal failure, hepatic insufficiency, glaucoma, head trauma, hypertension, and drug or alcohol abuse. Participants with learning disabilities, poor eyesight (severe myopia of -6 diopters or worse), who smoked more than 5 cigarettes a day, who were pregnant, or who were left-handed were also excluded.

### Drug administration

The study was conducted using, a randomized, double-blind cross-over design. Atomoxetine and placebo were administered in separate test sessions, randomly ordered across participants, spaced one week apart, and scheduled at approximately the same time of day. Atomoxetine (40 mg) was administered orally as a single, encapsulated pill, with a glass of water. 40 mg is the starting dose used in clinical practice for treatment of ADHD, and is a dose associated with limited side effects [52]. The placebo consisted of 125 mg of lactose monohydrate with 1% magnesium stearate, encapsulated, and identical in appearance to the drug. The pill was administered approximately 180 minutes before the participants began the task. The pharmacokinetics of atomoxetine indicate that peak drug effects should occur between 90 and 180 minutes, although there is considerable variability in this timing between extensive and poor metabolizers[30].

### Horizon task

We used a slightly modified version of the task described by Wilson and colleagues [4], in that we included a horizon 11 condition in addition to horizon 1 and 6 (note that this version of the task is almost identical to the version in the supplementary material of [4]). To keep the experiment duration down, our version was shortened to 120 games (in four blocks of 30 games each). Each game lasted either five, 10, or 15 trials, and these three conditions were presented interleaved in random order such that there were 40 games of each length. In each game, participants chose between two options, with each option rewarding the participant with a value between 1 and 100 points, sampled from a Gaussian distribution with a fixed standard deviation of 8 points, and centered on a distinct mean value for each option that was fixed within a given game, but changed between games. For each game, the mean of one option was set to either 40 or 60, and the mean of the second option was then set to be either 0, 5, or 10 points higher or lower than the value of the first. Both the identity of the 40/60 option and the difference in means were counterbalanced over the entire experiment.

Before the experiment, participants read standardized, detailed, on-screen instructions with examples illustrating that the means of the two options were constant over a given game, and that the variability in the options was constant over the entire experiment. Thus, participants were informed about the structure of the task. Participants were told that they should try to maximize their winnings by determining which option had the best value. The full text of the instructions is provided in Wilson et alsupplemental materials [4].

For each game, the choice and the value of each trial outcome remained visible along each side of the screen, so that participants could examine their record of decisions and outcomes and use that information to make current decisions (Fig 1). As participants made their decision on each trial *n*, the value appeared in the *n*th box beside the chosen option, and “XX” appeared in the *n*th box beside the other option.

We created two information conditions by controlling the first four trials of each game. These “forced-choice” trials could either give equal information about each option (two choices from each), or unequal information (three choices from one option, one choice from the other). This manipulation ensured that participants were exposed to a specified amount of information irrespective of the reward history on the first four trials, a crucial point given that otherwise option value would be correlated with amount of information because participants tend to choose the high value option more often. Thus on the first free choice (the fifth trial in each game), the difference in the number of times each option had been sampled (hence the difference in available information) had no effect on the difference in mean payout of that option.

The number of trials in a game determined the game’s horizon: the number of choices that could be made in a game, and thus the number of choices that could be used to explore the option values, and that would benefit from more accurate information about option values. After the first four forced choices, the horizon could be either 1, 6, or 11. We anticipated that the proportion of exploratory choices (choosing the less rewarding option) would increase for larger horizons, replicating Wilson and colleagues [4].

### Formal model

We analyzed behaviour on the first free-choice trial using the formal model proposed by Wilson and colleagues [4]. This model computes a value, *Q*^*i*^, for each option *i*, and makes probabilistic choices based on these values. The value of each option *Q*^*i*^ was computed as the weighted sum of the expected reward *R*^*i*^, information *I*^*i*^, spatial location *l*^*i*^, and random noise *n*^*i*^ (Note that we use a slight change in notation from our previous work, preferring now to use *l*^*i*^ for spatial location instead of *s*^*i*^ in [4]. This is to avoid confusion later on with the subscript *s* that we use to denote subject.):

$$Q^{i}=R^{i}+AI^{i}+Bl^{i}+\sigma n^{i}$$

where the free parameters *A*, *B* and *σ* correspond to the size of the information bonus, spatial bias and decision noise, respectively. So, the values of A and *σ* indicate the degree to which, in a given task condition and treatment condition, information seeking and decision noise contribute to the participant’s choice. If we assume that participants always choose the option with the highest value, then the probability of choosing option *i* is:

$$p^{i}=\frac{1}{1+\text{exp}\left(-\frac{\Delta R+A\Delta I+B\Delta l}{\sigma }\right)}$$

where *ΔR* = *R*^*i*^ − *R*^*j*^ is the difference in expected reward between the two options, (i.e. the difference in the mean reward of the two options experienced during the forced plays). *ΔI* = *I*^*i*^ − *I*^*j*^ is the difference in information between the two options. *ΔI* is defined such that *ΔI* = +1 if *i* was the more informative option (i.e., the option played once in the [1 3] condition), *ΔI* = −1 if *i* was the less informative option (the option played three times in the [1 3] condition) and *ΔI* was always zero in the [2 2] condition. *Δl* is the difference in location between the two sides and is defined as *Δl* = +1 if *i* was on the left and *Δl* = −1 if *i* was on the right.

By fitting this choice function to the participants’ data (i.e., the sigmoid choice curves), we were able to estimate the information bonus A, the bias B, and the magnitude of decision noise *σ*, separately for each participant, treatment, horizon and information condition.

### Model fitting

**Fig 4:**
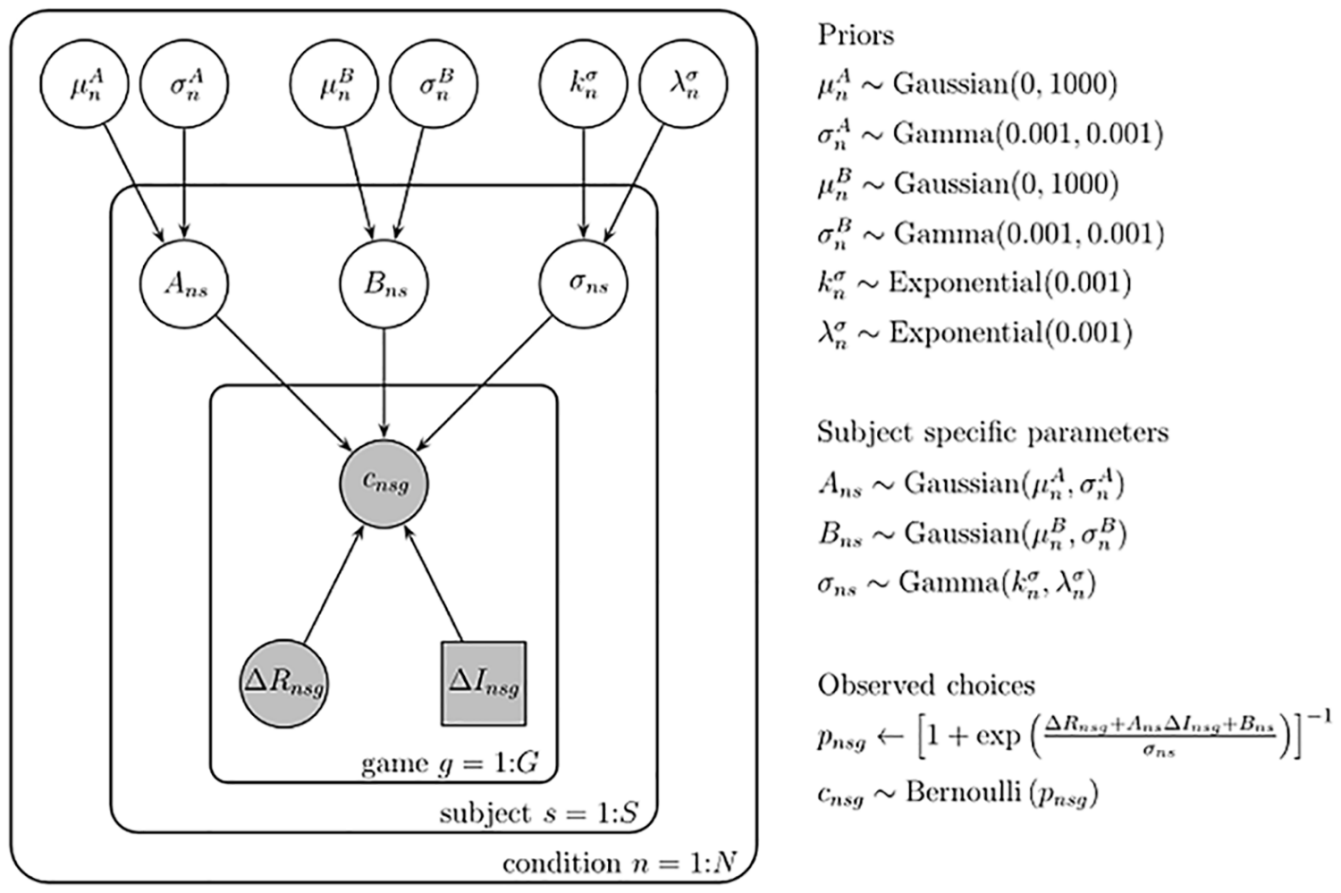
Graphical representation of the model. Hierarchical Bayesian models yield parameter estimates for each condition at both the subject level and the group level (Lee & Wagenmakers, 2014).

In our previous work [4], we used a maximum likelihood approach for model fitting. Here, we used a Bayesian approach (Fig 4), which is less vulnerable to over-fitting the data and is a more principled way of combining data from multiple subjects [53]. In particular, we assumed that each participant (*s*) had a different value of each parameter (*A*_*ns*_, *B*_*ns*_ and *σ*_*ns*_) in each condition (*n*), which was sampled from population-level distributions that were different for each parameter. In particular, the information bonus was sampled from a Gaussian distribution with mean $\mu_{n}^{A}$ and standard deviation $\sigma_{n}^{A}$:

$$A_{ns}∼\text{Gaussian}\left(\mu_{n}^{A},\sigma_{n}^{A}\right)$$

The spatial bias was also sampled from a Gaussian distribution:

$$B_{ns}∼\text{Gaussian}\left(\mu_{n}^{B},\sigma_{n}^{B}\right)$$

Because the decision noise, *σ*_*ns*_, could not be negative, we assumed that this was sampled from a Gamma distribution with shape parameter, $k_{n}^{\sigma }$, and scale parameter $\lambda_{n}^{\sigma }$, i.e.

$$\sigma_{ns}∼\text{Gamma}\left(k_{n}^{\sigma },\lambda_{n}^{\sigma }\right)$$

Finally, the hyperparameters, $\mu_{n}^{A}$, $\sigma_{n}^{A}$, $\mu_{n}^{B}$, $\sigma_{n}^{B}$, $k_{n}^{\sigma }$ and $\lambda_{n}^{\sigma }$ were themselves assumed to be sampled from prior distributions whose parameters we defined such that these priors were broad. Specifically,

$$\mu_{n}^{A}\text{ and }\sigma_{n}^{B}∼\text{Gaussian(0, 1000)}$$

$$\sigma_{n}^{A}\text{ and }\sigma_{n}^{B}∼\text{Gamma(0.001, 0.001)}$$

$$k_{n}^{\sigma }∼\text{Exponential(0.001)}$$

$$\lambda_{n}^{\sigma }∼\text{Exponential(0.001)}$$

To fit the model we used Markov Chain Monte Carlo (MCMC) sampling to generate samples from the posterior distribution over all model parameters, *A*_*ns*_, *B*_*ns*_, *σ*_*ns*_ $\mu_{n}^{A}$, $\;\sigma_{n}^{A}$, $\mu_{n}^{B}$, $\sigma_{n}^{B}$, $k_{n}^{\sigma }$ and $\lambda_{n}^{\sigma }$, given the behavioural data. The sampling was done using the JAGS package (http://mcmc-jags.sourceforge.net) via the Matlab interface matjags (http://psiexp.ss.uci.edu/research/programs_data/jags/). In all we ran four independent Markov chains. The first 500 samples from each chain were discarded to allow time for the sampling procedure to burn in and then 5000 samples were generated per Markov chain. To reduce the autocorrelation in our samples, only every 5^th^ sample in each chain was recorded, leaving us with 1000 samples per Markov chain and 4000 samples in all.

The model produced subject-level distributions of the values *A*_*ns*_, *B*_*ns*_ and *σ*_*ns*_ for each participant in each condition, as well as a set of population-level distributions of the parameters $\mu_{n}^{A}$, $\;\sigma_{n}^{A}$, $\mu_{n}^{B}$, $\sigma_{n}^{B}$, $k_{n}^{\sigma }$ and $\lambda_{n}^{\sigma }$ that described the subject-level distributions. To illustrate individual differences in behaviour we computed the mean values of the subject-level distributions, and to statistically test for effects of treatment, horizon, and information condition we took the median values of the population-level distributions (see below).

### Statistical analysis

Hierarchical Bayesian methods provide the most accurate estimates of individual participants’ parameters [54]. However, there is currently no software available for doing multifactorial robust Bayesian ANOVA [55]. Moreover, the frequentist ANOVA approach is not suitable for analyzing these subject-level estimates, because the population variance of subject-level Bayesian parameter estimates is underestimated (shrinking), and because the posterior estimates are not independent, violating the assumption of independence [56]. Thus, analyzing subject-level Bayesian parameter estimates with a standard ANOVA yields *p*-values that are biased toward the alternative hypothesis. In contrast to the subject-level parameter estimates, the standard frequentist approach does not bias *p*-values associated with the group-level parameter estimates (Boehm et al., under review). However, this approach introduces the challenge that repeated-measures ANOVAs cannot be calculated without referring to a subject-level error term. In light of these considerations, we analyzed our repeated-measures factorial design using a between-subject ANOVA of the group-level parameter estimates, which under plausible assumptions (positive correlations between repeated measures) provides a conservative estimate of the true *F* ratios and *p* values.
